# Novel vomputational methods for predicting epitopes of potent and broadly neutralizing HIV-1 antibodies

**DOI:** 10.1186/1742-4690-9-S2-P353

**Published:** 2012-09-13

**Authors:** MC Evans, A Paquet, P Phung, A Parikh, C Petropoulos, T Wrin, M Haddad

**Affiliations:** 1 Monogram Biosciences, South San Francisco, CA, USA

## Background

Recent efforts in HIV-1 vaccine design have focused on immunogens that evoke potent neutralizing antibody responses to a broad spectrum of viruses circulating worldwide. However, the development of effective vaccines will depend on the identification and characterization of the neutralizing antibody epitopes. Consequently, we developed bioinformatics methods to predict epitopes using corresponding genotypes and phenotypes generated using a highly sensitive and reproducible neutralization assay.

## Methods

Using 264 clonal envelope (gp120) sequences from a panel of multiclade HIV-1 viruses with matching neutralization titers to an array of neutralizing monoclonal antibodies (b12, PG9,16, PGT121 - 128, PGT130 - 131, PGT135 - 137, PGT141 - 145, and PGV04), we correlated IC_50_ titers with envelope mutations, and used this information to predict antibody epitopes. Structural patches were generated as amino acid groupings based on solvent-accessibility, diameter, atomic depth, and interaction networks within 3D envelope models. These patches were then evaluated as possible antibody targets by applying a boosted algorithm comprised of machine learning and statistical models. We identified residues with statistically significant correlation with IC_50_ titers as sites that impact neutralization sensitivity. Residues frequently occurring within the significant patches were mapped onto envelope structures as potential antibody binding sites.

## Results

Predicted epitopes were identified based on strong correlations with neutralization response to each antibody. Residues highly associated with the IC_50_ titers and patch clusters predicting neutralization response to these antibodies were located within V1/V2, and V3. The predicted response by the algorithm was highly concordant (>80%) with the neutralization sensitivity of all antibodies.

## Conclusion

We developed and applied computational methods to rapidly survey protein structures and identify epitope regions associated with neutralization response. This data mining algorithm can help identify immunological hotspots, and provide rapid and accurate insight into regions that are targeted by potent and broad neutralization responses. Studies are ongoing to confirm these novel epitopes.

